# Clinical study evaluating the efficacy and safety of Cilostazol as an adjuvant therapy to methotrexate on patients with rheumatoid arthritis

**DOI:** 10.1007/s10787-025-01782-2

**Published:** 2025-05-16

**Authors:** Samar M. Eldadamony, Sahar M. El-Haggar, Abdel Moaty A. Ali, Tarek M. Mostafa

**Affiliations:** 1 Clinical Pharmacy Department, Faculty of Pharmacy, Tanta University, Tanta, Egypt; 2 Department of Rheumatology, Rehabilitation and Physical Medicine, Faculty of Medicine, Mansoura University, Mansoura, Egypt

**Keywords:** RA, Methotrexate, Cilostazol, cAMP, DAS28-CRP

## Abstract

**Objective:**

This study aims to assess the safety as well as effectiveness of Cilostazol as a complementary therapy to methotrexate among individuals with active rheumatoid arthritis.

**Method:**

This study was a randomly allocated, double-blind, placebo-controlled parallel design involving 70 patients who were diagnosed with active rheumatoid arthritis. Participants were randomly assigned to two sets: the control group (*n* = 35) which received methotrexate "MTX" (7.5 mg IM weekly) plus placebo tablets twice daily and the Cilostazol group (*n* = 35), which received the same MTX" dose plus Cilostazol 50 mg twice daily for 3 months. Patients were assessed to determine the serum levels of C-reactive protein (CRP) nuclear factor kappa B (NF-κB), hemoxygenase-1 (HO-1), and cyclic adenosine monophosphate (cAMP). Disease Activity Score (DAS28-CRP), Multidimensional Health Assessment Score (MDHAQ), and morning stiffness duration were also assessed.

**Results:**

The Cilostazol group produced a significant improvement in cAMP level as compared to the control group (*P* = 0.001)**.** cAMP level showed a significant inverse correlation with DAS28-CRP (*r* = −0.336; *P* = 0.004). However, Cilostazol group produced non-significant improvements in the serum levels of the other biological markers (CRP, NF-κB, and HO-1), DAS28-CRP score, MDHAQ score, and morning stiffness duration as compared to the control group (P > 0.05). The implication of Cilostazol for patients with rheumatoid arthritis was tolerable and safe.

**Conclusion:**

The beneficial effect of Cilostazol on cAMP and the negative correlation between cAMP and DAS28-CRP could support its impact on the disease activity. Further research seems necessary to elucidate the mechanisms underlying the link between cAMP and disease activity.

**Trial registration:**

Clinical Trials.gov identifier: NCT05594680**,** The date of registration is: 30/10/2022.

## Introduction

Rheumatoid arthritis (RA) is an immune-mediated disorder marked by ongoing joint inflammation and articular destruction (Edilova et al. 2021). Tumor necrosis factor-α (TNF-α) and interleukin-1 beta (IL-1β), which are derived from phagocytes, are powerful triggers that promote growth of synovial cells around damaged cartilage. This leads to the ongoing, detrimental, and invasive spread of synovial tissues, which eventually causes joint erosion (He et al. 2020).

While the precise etiopathology of RA remains unknown, researchers hypothesized a range of genetic and environmental factors contribute to the pathogenesis of RA (Antunes Andrade et al. 2021). The receptor activator of the nuclear factor kappa B ligand (RANKL), a member of the tumor necrosis factor family produced by osteoblasts and bone marrow stromal cells, is required for the development and stimulation of osteoclasts, which play a pivotal role in the pathogenesis of bone deterioration in rheumatoid arthritis.

Heme oxygenase-1 (HO-1), which is recognized for protecting the cells from oxidative damage, may also have anti-inflammatory properties (Campbell et al. 2021). The synovial tissue obtained from RA patients, peripheral blood monocytes, and up-regulation of HO-1 in lipopolysaccharide (LPS)-treated synovial cell lines lead to a decrease in pro-inflammatory markers (Funes et al. 2020). The rise in intracellular cyclic adenosine monophosphate (CAMP)-coupled protein kinase A (PKA) catalyzation has been reported to stimulate the expression of the HO-1 gene in smooth muscle cells cultured from vascular tissue and in rat liver cells.

There is an opposite relationship between cAMP level in synovial fluid with the amount of synovial C-reactive protein (CRP) and interleukin-18 (IL-18) in RA patients. This discovery underscores the prospective advantages of cAMP-elevating medications in the treatment of RA (Xie et al. 2023). The primary course of therapy for RA patients is methotrexate (MTX), which inhibits neutrophil chemotaxis, reduces the growth of synovial fibroblasts, and modifies the production of superoxide anion and cytokines (García-González and Baker 2022). Methotrexate is a novel anti-proliferative medication used to treat cancer by preventing the production of pyrimidines and purines. Nevertheless, data quickly became available demonstrating that adenosine mediates a large portion of MTX's anti-inflammatory action (García-González and Baker 2022).

Methotrexate can boost the production of cAMP by activating A2A and A2B adenosine receptors through Gs proteins. The anti-inflammatory action of MTX is related to stimulation of adenosine A2A receptors (Darwish et al. 2022).

Cilostazol, an inhibitor of phosphodiesterase type III, significantly reduces inflammation by reducing nuclear factor kappa B (NF-κB) gene transcription through cAMP-dependent protein kinase activation (Sakamoto et al. 2018). Cilostazol was also reported to inhibit the proliferation of synovial cells among individuals with RA (Fangy et al. 2022). The anti-arthritic property of Cilostazol is attributed to the production of HO-1 which is linked to nuclear factor erythroid 2-related factor 2 (Nrf2) and cyclic AMP-dependent protein kinase stimulation (He et al. 0.^2020^). Cilostazol dramatically reduces Toll-like receptor 4 (TLR4) expression in synovial phagocytes derived from RA cases via inhibiting the Purine-rich box1 (PU.1) transcriptional activity (He et al. 2020; Carlaf and Brito 2020). Cilostazol prevents RANKL-induced osteoclastogenesis by blocking the purine-rich box1 linked receptor activator of nuclear factor κ B (PU.1-linked RANK) expression through Sirtuin 1 gene (SIRT1) stimulation, which in turn prevents osteoclast differentiation in the arthritic reaction (Niu et al. 2022). Also, Cilostazol attenuates inhibitor of nuclear factor kappa B (IκBα) destruction, nuclear factor kappa light chain enhancer of activated B cells p65 (NF-κB p65) nuclear translocation, which was linked to a decline in TNF-α as well as IL-1β synthesis (El Shazly et al. 2020). Furthermore, in a murine model of collagen-induced arthritis (CIA), Cilostazol treatment significantly reduced synovial inflammation and bone deterioration (Niu et al. 2022).

Because MTX's adverse effects are dose-dependent, medical implication of such drug necessitates the administration of low doses (Wang et al. 2018). Thus, we hypothesized that; a combined therapy of Cilostazol with MTX could provide a favorable therapeutic effect in individuals with rheumatoid arthritis. The simultaneous administration of low-dose MTX and Cilostazol could be an effective treatment strategy for mitigating joint deterioration and for attenuating of inflammation in RA by activating synovial fibroblasts' cAMP-dependent protein kinase and significantly reducing the production of cytokines, such as TNF-α, IL-1β, IL-6, and monocyte chemoattractant protein 1 (MCP1) in additive fashion (Kim et al. 2012).

## Patients and methods

### Research methodology and study population

This proof-of-concept randomized, double-blind placebo-controlled parallel trial included 70 patients with active rheumatoid arthritis who were diagnosed according to the American College of Rheumatology (ACR)/European League Against Rheumatism "EULAR, 2010" (Felson DT et al. 2011). The patients were recruited from Rheumatology, Rehabilitation and Physical Medicine Department, Mansoura University Hospital, Mansoura, Egypt. The blindness was maintained by the similarity between the placebo and Cilostazol tablets. The patients were allocated randomly into two groups: group 1 (control group; n = 35) which was given IM Methotrexate (7.5 mg weekly plus placebo tablet twice daily 2 h after meal for 3 months and group 2 (Cilostazol group; *n* = 35) which received IM Methotrexate (7.5 mg weekly) plus Cilostazol (Cilosort™, Multicare, Pharmaceutical, Egypt) 50 mg twice daily 2 h after meal for 3 months. The study protocol was approved from the Research Ethics Committee of Tanta University (Approval code: 35930\10\2) and the Medical Research Ethics Committee of Mansoura University (Approval code: MS.22.10.2163). The research followed the 1964 Declaration of Helsinki ethical criteria. The patients gave their written informed consented. The research was registered on ClinicalTrials.gov with an identification code: NCT05594680.

The inclusion criteria encompassed individuals diagnosed with active rheumatoid arthritis (RA) with 28-joint disease activity score (DAS-28) exceeds 2.6, age 18–60 years, and both genders. The exclusion criteria ruled out individuals with diabetes, cardiovascular conditions (e.g., arrhythmia, congestive heart failure), hematological diseases (severe anemia, coagulation disorders), and inflammatory conditions Additionally, individuals using low doses of aspirin, anticoagulants, antioxidants, and biological DMARDs, as well as those with renal and hepatic dysfunction, and those with hypersensitivity to the study medications were excluded. Pregnant and lactating women were also excluded.

### Methods

#### . History, clinical assessment, and anthropometric data collection

Medical history was taken, clinical assessment of morning stiffness duration and the presence of joint pain or swelling were done, demographic parameters (age, sex,) were collected, and anthropometric measurements were done including weight and height with subsequent calculation of body mass index (BMI) = [Weight (kg)] ÷ [Height^2^ (m^2^)].

#### Blood sample collection and assessment of biological markers

Before and 3 months following the intervention, 5 ml of venous blood was withdrawn from each patient between 8:30 am and 10:30 am after 12-h overnight fasting. Blood samples were placed in plain tubes and then centrifuged for 10 min at 3000 rpm. The biological markers were measured in the separated sera using enzyme-linked immune sorbent assay (ELISA) according to the manufacturer’s guidelines for assessing serum levels of human CRP (SunRed, China; Cat#201–12-1799), NF-κBp65 (SunRed, China; Cat#201–12-0667), HO-1 (DL-Develop, China; Cat#DL-HO-1-Hu), and cAMP (DL-Develop, China, Cat#DL-cAMP-Ge).

#### Disease activity assessment:

Disease activity was measured before and 3 months after treatment using the formula: DAS28-CRP = [0.56 √(tender joint count) + 0.28√(swollen joint count) + 0.36*ln (CRP + 1)] * 1.10 + 1.15. Disease activity was categorized as follows: high disease activity (≥ 5.1), low disease activity (≤ 3.2), and remission (< 2.6) (Wells et al. 2009).

#### Functional assessment

Before and 3 months after intervention, the validated Arabic Multidimensional Health Assessment Questionnaire (MDHAQ) that includes 14-items Health Assessment Questionnaire (HAQ) was implicated to assess function. The score of the HAQ Questionnaire is calculated from the mean of the sum of the responses of the items where each item is scored from 0–3, where 0 = without any difficulty, 1 = with some difficulty, 2 = with much difficulty, and 3 = unable to do (Aflaki et al. 2024). In addition, morning stiffness (MS) duration in minutes was also assessed.

#### Assessment of participants' compliance, and drug tolerability

Cilostazol and placebo were given monthly, and pill returned were counted to assess compliance. Participants were followed up weekly by phone calls and monthly through planned appointments to assess adherence and report drug-related side events. Standardized adverse effect checklists were implicated to report adverse effects.

### Primary and secondary outcomes

The main outcome is the variation in the biological marker serum concentration. The secondary outcome is the variation in DAS-28-CRP and MDHAQ scores.

### Sample size calculation

The sample size was determined using IBM^®^ SPSS^®^ Statistics version 28 software, assuming a 60% improvement in DAS-28 for the Cilostazol group and a 25% improvement for the group given the placebo, with a statistical power of 80% (Van Gestel et al.^1999^). A sample size of 31 patients per group has been suggested. In this context, a total sample size of 70 patients with 35 in each group is established as the initial target for the current study, assuming that the attrition rate is 10%.

### Statistical analysis

The data collected were organized using Microsoft^®^ Office Excel 2019 (Microsoft Corporation). Statistical analyses were performed using IBM^®^ SPSS^®^ Statistics version 28 (IBM Corp., Armonk, NY, USA). Graphical representations were created using GraphPad Prism version 6.01 (GraphPad Software, La Jolla, CA, USA). Data were assessed for normality using t Shapiro–Wilk test or the Kolmogorov–Smirnov test. For parametric data, paired *t* test was used to compare means within the same group, while unpaired t test was employed to compare means between two groups. Non-parametric data were analyzed using the Mann–Whitney *U* test for both intra-group and inter-group comparisons. Categorical data were evaluated using Chi-square test, and Fisher’s exact test was applied to analyze adverse effect reports. Results were expressed as mean ± standard deviation (SD), medians, range, and percentages, as appropriate. Statistical significance was set at p ≤ 0.05.

## Results

**Fig. 1:**
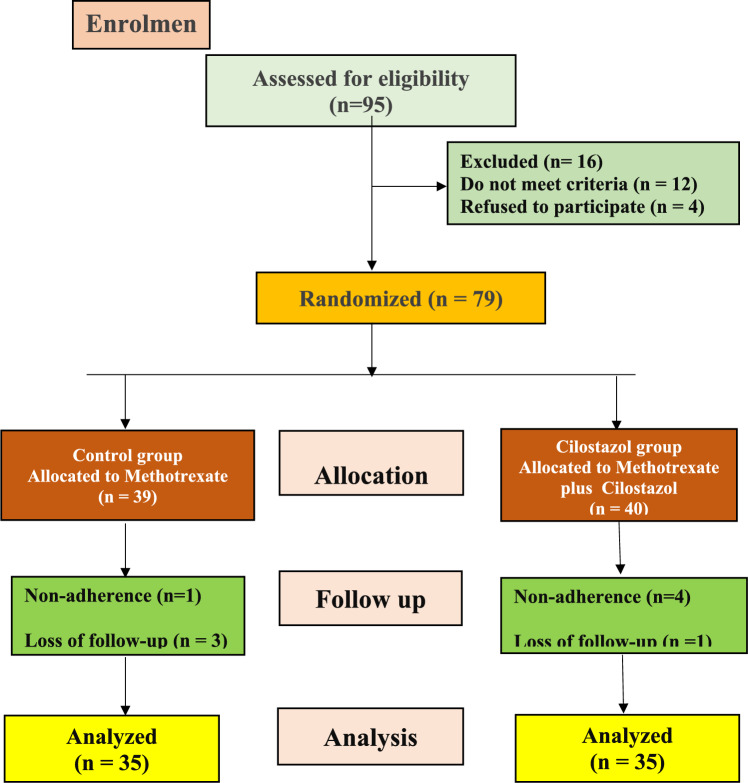
Participant flowchart

Figure 1 shows participant flowchart. Out of 95 RA patients assessed for eligibility, 16 were excluded owing to the study exclusion criteria (*n* = 12) or their refusal to participate (*n* = 4). Thus, 79 patients were randomly divided into two groups. During the follow-up period, 9 patients from the two groups were excluded due to non-adherence or loss of follow-up. The final analysis included 70 patients (35 patients per group).

### Demographic characteristics and other baseline data

Table 1 shows that the two study groups had comparable demographics, illness duration, rheumatoid factor status, and smoking habit (P > 0.05).

**Table 1:** Demographic, anthropometric, and other baseline data for the two study groups

Variables			Groups							P value
			Control group (*n* = 35)				Cilostazol group (*n* = 35)			
Age (years)		Range	22	–		60	26	–	60	0.903
		Mean ± SD	44.886	±		8.874	45.143	±	8.799	
			N			%	N		%	
Sex		Male	2			5.71	7		20	0.074
		Female	33			94.29	28		80	
Smoking		Yes	3			8.57	4	0	11.43	0.690
		No	32			91.43	31		88.57	
Disease duration (years)		Range	2	–		16	2	–	20	0.736
		Mean ± S.D	7.400	±		4.414	7.743	±	4.046	
MTX dose (mg)	Range		0.5—1				0.5—1			0.690
	Mean ± S.D		0.843 ± 0.205				0.863 ± 0.177			
BMI (kg/m^2^)	Range		23.9–40				22.5–39			0.707
	Mean ± S.D		28.957 ± 3.990				28.609 ± 3.742			
RF		Positive	N			%	N		%	1.000
			30			85.71	30		85.71	
		Negative	5			14.29	5		14.29	

Data are presented as mean ± standard deviation; number and percent

*MTX* Methotrexate, *BMI* Body mass index, *mg* milligram, *Kg* kilogram, *m*^*2*^ meter square, *RF* rheumatoid factor

Significance level was set at *p* value < 0.05

### Effect of treatment on assessed biomarkers

The laboratory parameters at baseline showed non-significant difference between the two groups (p > 0.05), as presented in Table 2. Three months post-intervention and as compared to baseline values, the control group exhibited a significant reduction in the serum levels of C-reactive protein "CRP" (*P*_1_ = 0.024) and cyclic adenosine monophosphate "cAMP" (*P*_1_ = 0.001), which was associated with non-significant changes in Hemoxygenase-1 "HO-1" (*P*_1_ = 0.216) and nuclear factor kappa B "NF-κB" (*P*_1_ = 0.701). The Cilostazol group produced a significant decrease in the serum concentration of CRP (P_1_ = 0.016) and a significant increase in cAMP serum level (*P*1 = 0.049) which was associated with non-significant changes in the serum levels of Hemoxygenase-1 "HO-1") (*P*_1_ = 0.097), and nuclear factor kappa B "NF-κB" (P_1_0.801). Three months post-intervention, the comparison of the two study groups revealed that, Cilostazol group showed a significant increase in the serum levels of cAMP (*P*_2_ = 0.001) as compared to the control group. However, there were non-significant variations between the two groups regarding the serum levels of CRP (*P*_2_ = 0.330), HO-1 (*P*_2_ = 0.902) and NF-κB (*P*_2_ = 0.080) as postulated in Table 2.

**Table 2:** The serum levels of biological markers for the two study groups

Variables		Control group (*n* = 35)		Cilostazol group (*n* = 35)			*P*_2_ value
		Before treatment	After treatment	Before treatment		After treatment	
CRP (mg/L)	Range	7–49	6–65	6–96		6–45	0.330
	Mean ± SD	21.537 ± 10.849	17.557 ± 13.482	20.260 ± 18.128		14.569 ± 11.953	
P_1_ value		0.024*		0.016*			
HO-1 (ng/ml)	Range	19–44.2	19.8–41.9	19.1–42.9	18.1–40.4		0.902
	Mean ± SD	31.843 ± 6.688	30.911 ± 6.429	32.157 ± 5.849	30.723 ± 6.365		
P_1_ value		**0.216**		**0.097**			
cAMP (nmo/L)	Range	0.04–3.4	0.03–2.5	0.02–2.2	0.01–2.3		0.001*
	Median )IQR(	0.39 (0.24–0.83)	0.19 (0.11–0.31)	0.22 (0.19–0.4)	0.44 (0.24–1.16)		
P_1_-Value		0.001*		0.049*			
NF-κB (ng/ml)	Range	0.09–2.3	0.4–1.3	0.09–3.3	0.35–2.9		0.080
	Mean ± SD	0.927 ± 0.390	0.905 ± 0.233	1.118 ± 0.578	1.091 ± 0.573		
P_1_ value		**0.701**		**0.801**			

Data are expressed as mean ± SD, range, median, *IQR* Inter Quartile Range, *CRP* C-reactive protein, *mg/L* milligram per liter, *Ho-1* Hemoxygenase-1, *ng/ml* nanogram per milliliter, *cAMP* cyclic adenosine monophosphate, *nmol/L* nanomole per liter, *NF-κB* Nuclear factor kappa B

P_1_ value: difference within the same group (before versus after treatment)

P_2_ value: difference between the two groups after treatment

Significance level was set at *p* < 0.05

^*^ Significant difference

### Effect of treatment on clinical and functional assessments

The clinical and Functional features for all participants in the two study groups at baseline showed no significant difference (p > 0.05). The data obtained with this study demonstrated that, 3 months after intervention and as compared to baseline data, the control group showed non-significant variation in DAS28-CRP score (P_1_ = 0.308). However, this group showed significant improvement in MDHAQ score (*P*_1_ = 0.005) and morning stiffness duration (P_1_ < 0.001). Three months after intervention and as compared to baseline data, Cilostazol group produced a significant decrease in DAS28-CRP score (*P*_1_ < 0.001), MDHAQ score (P_1_ < 0.001), and morning stiffness duration (*P*_1_ = 0.001) as demonstrated in Table 3. The comparison of the two study groups 3 months after intervention revealed that there were non-significant variations regarding DAS28-CRP score (*P*_2_ = 0.574), MDHAQ score (*P*_2_ = 0.867), and morning stiffness duration (*P*_2_ = 0.804) as postulated in Table 3.

**Table 3:** Clinical and functional assessments for the two study groups

Variables		Control group (*n* = 35)			Cilostazol group (*n* = 35)		*P*_2_ value
		Before treatment		After treatment	Before treatment	After treatment	
DAS28-CRP	Range	2.54–6.4		1.5–6.8	3.33–6.67	2.67–6.1	0.574
	Mean ± SD	4.569 ± 0.839		4.341 ± 1.361	4.823 ± 0.811	4.186 ± 0.883	
P_1_ value		**0.308**			< 0.001*		
MDHAQ	Range	1.67–10		2–8	2–8	2–8	0.867
	Mean ± SD	4.909 ± 1.784		4.258 ± 1.478	4.978 ± 1.478	4.318 ± 1.497	
P_1_ value		0.005*			< 0.001*		
Morning stiffness (hours)	Range	0.17–1.5	0.08–1		0.17–1.5	0.08–1	0.804
	Mean ± SD	0.776 ± 0.346	0.574 ± 0.410		0.769 ± 0.338	0.550 ± 0.386	
P_1_-Value		< 0.001*			0.001*		

Data are expressed as mean ± SD and rang

*DAS28-CRP* Disease Activity Score-28 using C- reactive protein, *MDHAQ* Multidimensional Health Assessment Questionnaire

P_1_ value: difference within the same group (before versus after treatment)

P_2_ value: difference between the two groups after treatment

Significance level was set at *p* < 0.05

^*^ Significant difference

### Correlation analysis

**Fig. 2:**
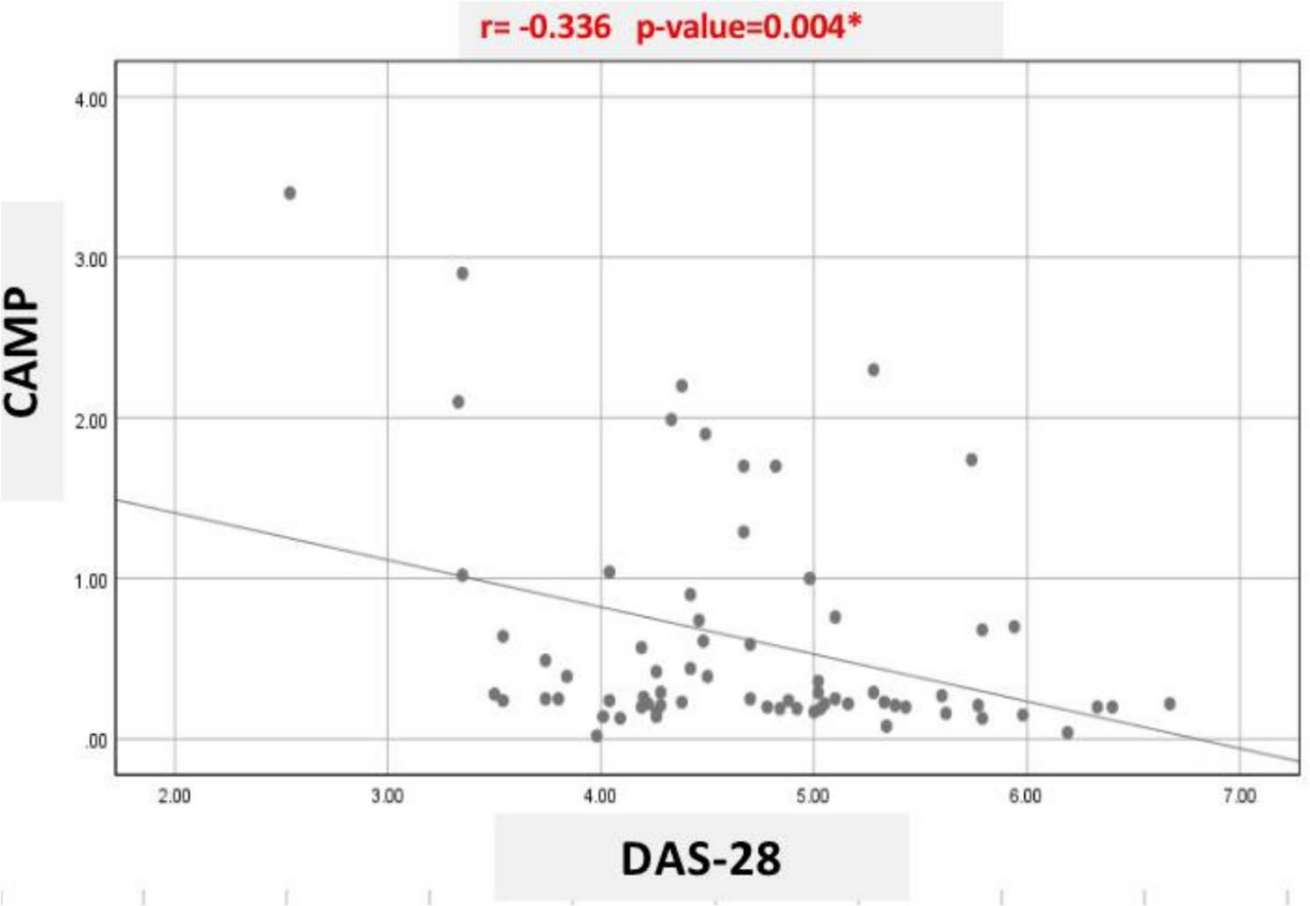
The correlation between cAMP and DAS-28

The correlation analysis conducted on the variables in the Cilostazol group post-treatment showed the existence of significant negative correlation between cAMP values and the DAS28 score (r = −0.336; *P* = 0.004) as demonstrated in Fig. 2.

### Drug safety and tolerability

**Fig. 3:**
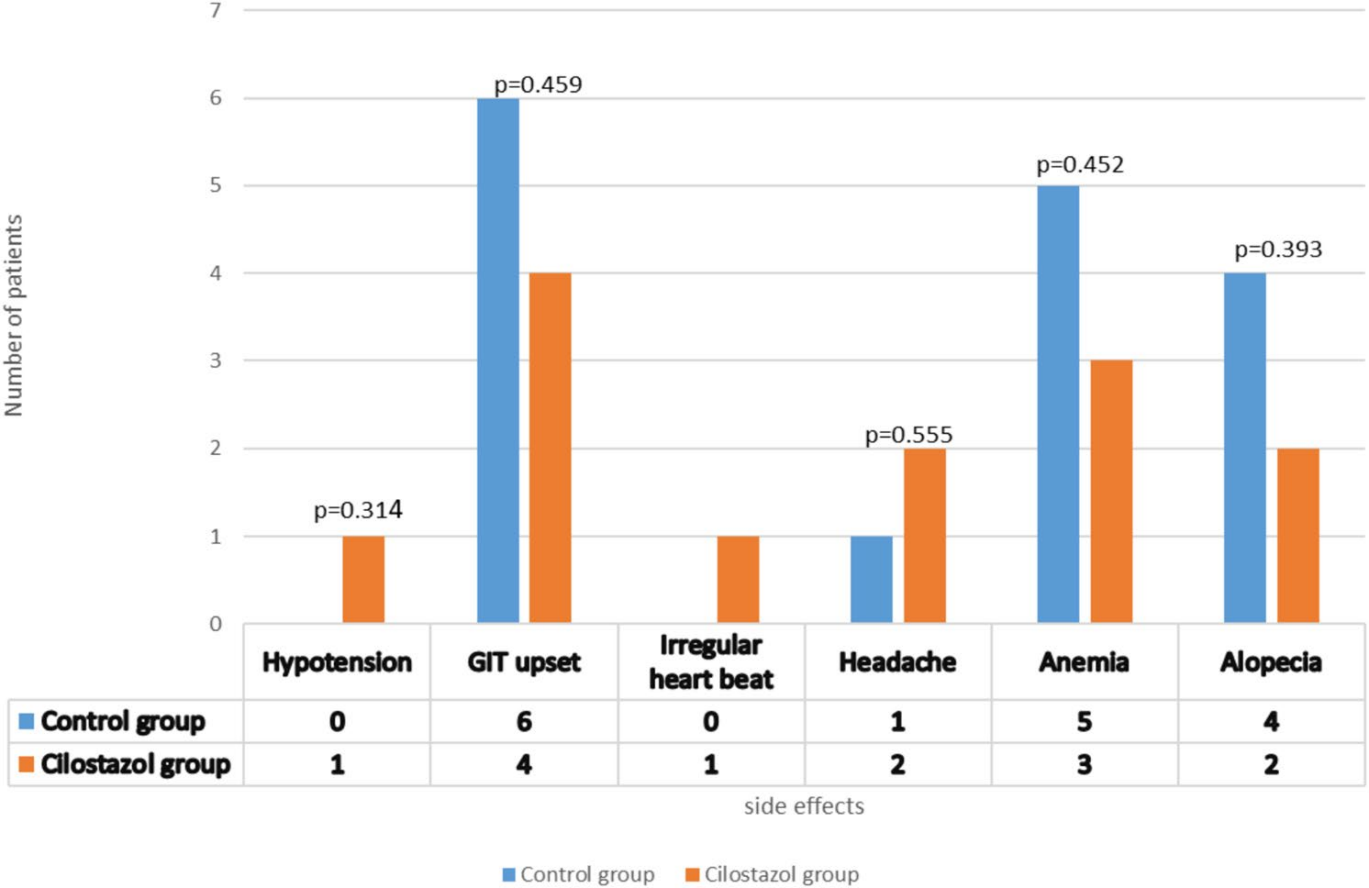
The reported side effects for the two study groups

In the control group, six patients (17.1%) suffered from stomach upset, one patient (2.9%) had headache, five patients (14.3%) developed anemia, and four patients (11.4%) developed alopecia. In the Cilostazol group, four patients (11.4%) showed stomach upset, two patients had headache (5.7%), three patients developed anemia (8.6%), two patients got alopecia (5.7%), one patient abnormal heartbeat (2.9%), and one patient showed hypotension (2.9%). No significant difference in the reported adverse effects was detected between groups (*P* > 0.05). In conclusion, both control and Cilostazol groups have comparable safety and tolerability as demonstrated in Fig. 3.

## Discussion

Methotrexate is a popular DMARD which is commonly implicated for the management of rheumatoid arthritis. Secondary to its adverse effects, including stomach pain, baldness, mouth ulcerations, and cytopenia, its usage is restricted despite its disease-reducing efficacy (Solomon et al. 2020).

The present research aimed at examining the effectiveness and safety of Cilostazol, a PDE inhibitor, as an adjunct therapy to methotrexate for patients with active rheumatoid arthritis. The two research groups were statistically similar at baseline; thus, any differences following treatment are attributed to the study medications, and hence are not related to individual variations.

Approximately 80% of the participants in the two research groups were women which supports the hypothesis that disease is more common among women (Angum et al. 2020).

Rheumatoid arthritis (RA) patients have elevated CRP levels, which indicate systemic inflammation. C-reactive protein (CRP) regulates the immune system and promotes atherogenesis via inflammatory mechanisms (Pope et al. 2019).

The present research elucidated that 3 months following intervention, both the control group (MTX alone) and the Cilostazol group (MTX plus Cilostazol) produced significant improvements in CRP level, MDHAQ score, and morning stiffness (MS) duration as compared to baseline values. Only Cilostazol group produced a statistically significant improvement in DAS28-CRP score as compared to baseline value. However, the difference between the two study groups at the end of intervention remains statistically non-significant; it seems clinically important, since Cilostazol group showed much lower CRP level, DAS28-CRP score, and morning stiffness duration than the control group. The lack of statistically significant variations between the two research groups comes in confliction with a previous preclinical study showed that Cilostazol has anti-inflammatory activity (de Oliveira et al. 2022).

High blood cAMP levels seem very important, because cAMP is considered a major intracellular second messenger that has anti-inflammatory and tissue-protective effects (Pacini et al. 2022). The immune-modulatory role of cAMP was demonstrated by other authors who reported an inverse association between the amount of cAMP in synovial fluid and the levels of CRP and IL-18 (Morovic-Vergles et al. 2008). This formerly reported finding suggests that cAMP-elevating drugs could be helpful in the treatment of RA. In the current study, 3 months post-treatment the control group exhibited a significant drop in cAMP level as compared to baseline data. On the other hand, the Cilostazol group showed significantly higher level of cAMP as compared to its baseline value and the control group. The significant variation in the cAMP level between the two groups in the favor of Cilostazol group supports the idea that adding Cilostazol to MTX for rheumatoid arthritis could provide pharmacologic and clinical outcomes (Kim et al. 2012).

Haem oxygenase (HO-1), an inducible haem-degrading enzyme, is hypothesized to have antioxidant properties. Recent research showed that HO-1 has also anti-inflammatory properties (Ryter et al. 2021). The nuclear factor kappa B (NF-Kb) pathway, which expresses several pro-inflammatory genes, such as cytokines, chemokines, and adhesion molecules, is also regarded as a classic pro-inflammatory signaling system (Zhang et al. 2021). A previous study revealed an increase in intracellular cyclic adenosine monophosphate (cAMP) coupled protein kinase A (PKA) which in turn stimulates hepatic HO-1 gene expression (Park et al. 2010). In the present study, neither the combination therapy (MTX plus Cilostazol) nor MTX alone resulted in substantial alterations in the levels of both heme oxygenase-1 (HO-1) and nuclear factor kappa B (NF-κB). Furthermore, there were no significant variations between the two study groups regarding their effects on both HO-1 and NF-kB levels. This contradiction between our findings and the finding reported by Park et al., 2010 could be explained by the limited sample size, the short treatment duration, the implication of the low dosage of Cilostazol (50 mg twice daily), and the variation in the study designs.

During the current work, we noted a negative and significant association between cAMP level and DAS28-CRP score which suggests that Cilostazol through modulation of cAMP concentration could provide a favorable impact on disease activity in patients with RA. Since there was non-significant impact of Cilostazol on the assessed inflammatory markers, further research is still needed to determine the causality and the real mechanisms underlying the association between cAMP and the disease activity during RA.

The reported side effects revealed that hypotension and irregular heartbeat were not common side effects for Cilostazol. Our findings are consistent with a previous finding which reported that Cilostazol rarely causes blood pressure disturbance (Kherallah et al.^ 2022^). Additionally, 4 out of 35 patients in MTX/Cilostazol group developed a statically non-significant gastrointestinal upset, which comes in contradiction with other findings demonstrated that Cilostazol may cause gastrointestinal disturbances (Kherallah et al. 2022). The comparison between the two groups revealed the absence of significant difference regarding the reported adverse effects which proves safety and tolerability of Cilostazol.

The strength points of the current research include its design as a randomized double-blind placebo-controlled parallel study. However, the current work has some drawbacks involving small sample size, short intervention duration, and the implication of fixed dose of Cilostazol. Therefore, further large-scale and more longitudinal studies are still required.

## Conclusion

The findings from the current study indicate that the addition of Cilostazol to methotrexate provoked a significant increase in cAMP level as compared to the methotrexate alone and cAMP level showed a significant negative correlation with DAS28-CRP. However, methotrexate plus Cilostazol did not provide significant improvements in the assessed biological markers, DAS28-CRP, MDHAQ score, and morning stiffness duration as compared to methotrexate mono-therapy. Additional exploration and long-term studies are required.

## Data Availability

The material may be acquired from the author upon request that is reasonable.
